# Exploring the Untapped Potential of Pine Nut Skin By-Products: A Holistic Characterization and Recycling Approach

**DOI:** 10.3390/foods13071044

**Published:** 2024-03-28

**Authors:** Agata Nolasco, Jonathan Squillante, Salvatore Velotto, Giovanni D’Auria, Pasquale Ferranti, Gianfranco Mamone, Maria Emanuela Errico, Roberto Avolio, Rachele Castaldo, Lucia De Luca, Raffaele Romano, Francesco Esposito, Teresa Cirillo

**Affiliations:** 1 Department of Agricultural Sciences, University of Naples “Federico II”, Via Università, 100, 100-80055 Portici, NA, Italy; 2 Department of Promotion of Human Sciences and the Quality of Life, University of Study of Roma “San Raffaele”, Via di Val Cannuta, 247-00166 Roma, Italy; 3 Institute of Food Science, National Research Council, 83100 Avellino, Italy; 4 Institute for Polymers Composites and Biomaterials-National Research Council of Italy (IPCB-CNR), Via Campi Flegrei 34, 80078 Pozzuoli, NA, Italy

**Keywords:** pine nuts, integument, recycling, agri-food waste, circular economy

## Abstract

The increasing population, food demand, waste management concerns, and the search for sustainable alternatives to plastic polymers have led researchers to explore the potential of waste materials. This study focused on a waste of pine nut processing referred to in this paper as pine nut skin. For the first time, its nutritional profile, potential bioactive peptide, contaminants, and morphological structure were assessed. Pine nut skin was composed mainly of carbohydrates (56.2%) and fiber (27.5%). The fat (9.8%) was about 45%, 35%, and 20% saturated, monounsaturated, and polyunsaturated fatty acid, respectively, and Omega-9,-6, and -3 were detected. Notably, oleic acid, known for its health benefits, was found in significant quantities, resembling its presence in pine nut oil. The presence of bioactive compounds such as eicosapentaenoic acid (EPA) and phytosterols further adds to its nutritional value. Some essential elements were reported, whereas most of the contaminants such as heavy metals, polycyclic aromatic hydrocarbons, rare earth elements, and pesticides were below the limit of quantification. Furthermore, the in silico analysis showed the occurrence of potential precursor peptides of bioactive compounds, indicating health-promoting attributes. Lastly, the morphological structural characterization of the pine nut skin was followed by Fourier Transform Infrared and solid-state NMR spectroscopy to identify the major components, such as lignin, cellulose, and hemicellulose. The thermostability of the pine nut skin was monitored via thermogravimetric analysis, and the surface of the integument was analyzed via scanning electron microscopy and volumetric nitrogen adsorption. This information provides a more comprehensive view of the potential uses of pine nut skin as a filler material for biocomposite materials. A full characterization of the by-products of the food chain is essential for their more appropriate reuse.

## 1. Introduction

### 1.1. Valorization of Food By-Products

By-products are generated in various industries, often discarded without recognizing their potential for reuse. The concept of the circular economy emphasizes the importance of reevaluating waste, particularly in the food sector. According to the Food and Agriculture Organization (FAO), global food loss and waste amount to approximately 1.6 billion tons annually, negatively impacting food security, the economy, and the environment [1]. With the projected world population reaching 9.1 billion by 2050, food production needs to increase by 70% to meet the growing demand [2]. Currently, about half of habitable land and over 70% of freshwater resources are utilized for global food production, leading to emissions of 23–34% of global anthropogenic greenhouse gases [3,4,5] and the depletion of Earth’s natural resources [6]. In light of this, reducing losses and waste along the food supply chain serves a dual purpose: minimizing the environmental impact of the food system and obtaining new products.

By-products from food processing can be valuable ingredients or serve as alternative food sources, enabling the production of functional and novel foods [7,8,9,10]. For example, the coffee bean generates substantial by-products during the processing phases. Recent studies have demonstrated that these by-products contain high-value compounds such as proteins, polysaccharides, fibers, and phenolic compounds [11,12,13]. Additionally, they are of interest for extracting the bioactive molecules used in pharmaceuticals, as well as food and non-food applications (e.g., cosmetics, industry) [14,15,16,17]. By-products from the industrial supply chain, such as apple pulp and citrus peels (lemon, lime, orange, and grapefruit), serve as sources of pectin, commonly used for preparing gelling food products [18]. Pectin extraction can also be performed using many other food industry by-products, thereby valorizing agro-industrial waste [19,20,21,22,23]. The properties of pectins find applications in various industries and nutraceuticals [24,25], including the development of biodegradable food packaging and edible coatings to enhance foods’ shelf life [26,27,28,29]. Besides pectins, other polysaccharides like chitosan and cellulose, as well as proteins, lipids, and carbohydrates, are utilized for edible coatings [30,31]. Moreover, the bioactive properties of these natural compounds confer antioxidant, antibacterial, and antifungal characteristics to the packaging materials [32,33,34]. The mismanagement of plastic waste and concerns about chemical migration into the environment and food have spurred research into natural, innovative, and sustainable materials [30,35].

In the agri-food sector, the production of nuts and dried fruits results in a significant amount of by-products. Global nut production reached approximately 4.6 million tons in the 2019/2020 season [36]. Almonds and walnuts constituted the majority, accounting for 31% and 21% of the global share, respectively, followed by cashews (17%), pistachios (14%), and hazelnuts (12%). The remaining 5% encompassed pecans, macadamias, Brazil nuts, and pine nuts. Nut processing generates by-products such as nut shells and integuments. While the latter is less bulky and cumbersome, it poses management challenges for companies. Recent studies have examined walnut shells, a lignocellulosic agricultural by-product that can be used as a bio-absorbent material for wastewater purification [37] or as a basis for biodegradable packaging [38]. Another by-product of walnut processing is the green hull, the fleshy and pulp-rich mesocarp of the fruit, which contains bioactive compounds with antioxidant, anti-inflammatory, and antibacterial properties [39,40]. By-products from hazelnut and pistachio processing are also utilized for isolating and purifying bioactive molecules with antioxidant functions [41,42]. Notably, understanding the composition of by-products derived from nuts, encompassing their nutraceuticals and contaminants, is crucial. Nutraceuticals and food ingredients from nuts may be contaminated with various substances such as pesticides, heavy metals, mycotoxins, and other chemical contaminants, which can pose risks to consumer health [43,44,45]. These contaminants can be found in nutraceuticals, which often contain modified or unmodified whole food, plant extracts, purified phytochemicals, or a combination of different phytochemicals [46]. Therefore, it is crucial to ensure the safety and quality of these products to prevent potential health hazards. Thus, it is not uncommon to identify compounds or functions with promising possibilities for reusing these by-products, although there are still many gaps in knowledge in this area. This study contributes to the research and characterization of previously overlooked waste products. An example is the waste generated from pine nut processing, which constitutes a small part of the dried fruit market and has limited knowledge available.

### 1.2. The Pine Nut Chain and Its Integument By-Product

Pine nuts are highly nutritious seeds that are recognized and consumed worldwide. While accounting for less than 1% of the worldwide nut market, pine nuts have experienced a notable surge in interest [47]. In the 2020/2021 season, global pine nut production reached 38,175,000 kg, marking a 48% increase from the previous season. The International Nuts and Dried Fruit (INC) report identifies Russia (31%), China (28%), and North Korea (23%) as the top producers, while Turkey, Italy, Spain, and Portugal are the main Mediterranean producers, contributing a combined total of 3% [36].

In Europe, pine nuts are highly sought after and command premium prices, with retail prices reaching up to 100 euros per kilogram [48]. The high production costs in Europe can be attributed to various factors. 

The pine nut production process involves the preliminary drying of pinecones, coarse grinding, sieving, and heating the nuts in a rotary oven. Subsequent steps include further selection, washing, a final drying, and brushing. During this final processing stage, the thin integument that covers the pine nuts is released as a by-product.

In line with the Sustainable Development Goals (SDGs), the International Nuts and Dried Fruit (INC) organization has been evaluating more sustainable production and trade practices, with a focus on improving waste management [45]. Given the high market demand, cost, and nutritional value of pine nuts, it is particularly important to adopt an integrated supply chain approach that facilitates the reuse of waste materials. 

Exploring the potential of by-products in the food industry offers promising prospects across various sectors. From agriculture to manufacturing, repurposing these overlooked resources can drive innovation and foster sustainable practices. Recent studies within the nut industry have shed light on the valuable properties of almond skins, pistachio skins, and hazelnut skins, once considered to be mere waste products. Research reveals that these nut skins are abundant in antioxidants, positioning them as ideal candidates for nutraceutical development [49]. Integrating by-products like pine nut skin (PNS) into industrial processes not only minimizes waste but also opens avenues for new revenue streams and the creation of products with enhanced functionalities. For instance, PNS could feature in the production of biodegradable packaging materials, meeting the rising demand for eco-friendly alternatives in the packaging sector. Furthermore, its bioactive attributes make it a compelling option for nutraceutical applications, contributing to the expanding health and wellness market. Embracing such practices not only aligns with sustainable development goals but also underscores responsible resource management within industries.

In this regard, this study is innovative and pioneering because it focuses on the characterization and enhancement of the integument (skin) that is separated during the final phase of drying and considered to be a waste. This by-product from the nut industry has remained largely unexplored and understudied until now, despite its significant potential for various applications. To this end, a complete characterization of the integument was carried out to explore its characteristics and potential uses. This includes assessing its nutritional profile and ensuring food safety by analyzing its absence of contaminants and its potential for reuse as a food ingredient. Furthermore, evaluating its peptide composition and biological activities can determine its suitability for nutraceutical applications. Lastly, emphasizing its suitability for various industrial applications, the research delves into the morphological and structural characteristics of PNS using advanced techniques such as FT-IR spectroscopy, solid-state CP/MAS 13C NMR spectroscopy, TGA, SEM, and nitrogen adsorption. 

The overarching goal is to provide valuable insights into how PNS could be utilized in functional foods, nutraceuticals, and potentially in the industrial sector, aligning with sustainable and resource-efficient practices. Through this focused investigation, the manuscript aims to contribute to the growing knowledge of agricultural by-products, paving the way for their innovative and practical applications. 

## 2. Materials and Methods

### 2.1. Pine Nut Skin Sample

The pine nut skin (PNS) was supplied by the company Ciavolino, located in Naples, Italy. The company, founded in 1950, mainly processes pinecones from *Pinus pinea* trees cultivated in the Vesuvian area. These particular pine nuts are renowned for their elongated shape and ivory-white color. Throughout the production and processing stages, Ciavolino ensures that the organoleptic and nutritional properties of the pine nuts remain unaltered. The company utilizes a dry-cleaning technique that effectively minimizes bacterial contamination while preserving the beneficial attributes of the nuts. To maintain the quality of the PNS samples, they were stored in a dark environment at a temperature of 20 °C and analyzed within a three-day timeframe.

### 2.2. Characterization of Nutritional Profile

A nutritional profile and wholesomeness assessment were conducted on the pine nut integument samples. 

The comprehensive procedures for the protein fraction, carbohydrate, total fiber, and fat content can be found in the study conducted by [13]. The authors provide detailed information on the methods employed for the extraction of these components.

#### 2.2.1. Protein Fraction

The protein fraction analysis followed the Kjeldahl procedure, a method that quantifies the nitrogen content in a sample [50]. The preparation of pine nut skin samples was performed in triplicate.

#### 2.2.2. Carbohydrates

A 10 g sample of finely ground PNS was extracted using a solution of acetonitrile and water in a 60:40 ratio, and placed in an ultrasonic bath for 30 min. After the extraction process, the sample underwent stirring for an additional three hours. The separation and determination were conducted through high-performance liquid chromatography (HPLC) employing a dedicated chromatographic column. Detection was performed using a UV detector.

#### 2.2.3. Total Dietary Fiber

To determine the quantity of the total dietary fiber, an enzymatic digestion process was utilized with the Megazyme Integrated Total Dietary Fiber Assay kit [51]. This method involves the application of heat-stable alpha-amylase, protease, and amyloglucosidase [50]. Subsequently, ethanol precipitation was conducted, followed by filtration, and the drying of the resulting residue. The quantity of total dietary fiber is determined by the mass left after the deduction of the residual ash and protein content.

#### 2.2.4. Total Fat

The determination of total fat was carried out using the Soxhlet method, adhering to the official procedure outlined in the ISTISAN report 96/34 [50]. To determine the total fat content in the PNS, a cellulose extraction thimble was filled with 5 g of PNS powder and inserted into the Soxhlet apparatus. The PNS underwent a 3 h extraction using petroleum ether, followed by the removal of the solvent using a rotary evaporator. The resulting residue was then dried at 100 °C for 1 h. Finally, the weight of the extracted sample indicated the total fat content.

#### 2.2.5. Triacylglycerol Determination

The determination of triacylglicerols (TAGs) was carried out following the method used by [52] with some modifications. A 0.5% solution of fat in hexane was prepared, vortexed for a few minutes, and 1 μL of the solution was injected into an Agilent Technologies 6890N gas chromatograph equipped with a programmed temperature vaporizer (PTV), a flame ionization detector (FID) and a capillary column (30 m × 0.25 mm i.d.; film thickness 0.10 μm) RTX-65 TG (Restek Corp., Bellefonte, PA, USA). The operating conditions were: PTV at 100 °C for 0.10 min, with an increase of 500 °C/min up to 380 °C for 5 min; the oven temperature was set at 80 °C for 1 min, followed by an increase of 30 °C/min to 240 °C for 0.1 min and then 4 °C/min to 360 °C for 5 min. The gas carrier was helium with a constant flow of 2 mL/min.

TAGs were identified through the comparison of their retention times with a certified anhydrous milk-fat CRM 519 reference provided by the Community Bureau of Reference (Commission of the European Communities, Brussels, Belgium). This reference enabled the calculation of response factors (Rfs), facilitating the conversion of peak areas into weight percentages.

#### 2.2.6. Fatty Acid Determination

Fatty acid methyl esters (FAMEs) concentrations were measured following transesterification using gas chromatography. The profile of fatty acids was assessed with a gas chromatograph equipped with an FID detector (Agilent Technologies 6850 Series II, Santa Clara, CA, USA) post-transesterification. The GC method of [53], with modification, was used. Briefly, 1 μL of the solution containing the FAMEs was injected into gas chromatograph equipped with a capillary column (100 m × 0.25 mm inner diameter, film thickness of 0.20 μm) with a polystationary phase (90% biscyanopropyl/10% cyanopropylphenyl siloxane) (Supelco, Bellafonte, PA, USA), hydrogen flame ionization detector (FID), and a programmed temperature vaporizer (PTV). Helium with a flow rate of 1 mL min^−1^ was used as gas carrier. The oven temperature program was as follows: 140 °C × 5 min, a 4 °C min^−1^ ramp to 175 °C for 20 min, and a 3 °C min^−1^ ramp to 240 °C for 20 min. The chromatogram peaks were identified using an external 37-component standard (Supelco, Bellefonte, PA, USA). The results are expressed as relative % values.

#### 2.2.7. Wax Determination

The analysis of the waxes was carried out by the method proposed by [54], with modifications. An SPE Chromabond 1000 mg silica gel column (Macherey-Nagel, Duren, Germany) was rinsed with 6 mL of carbon tetrachloride. Then, 20 mg of fat was weighed in a test tube, and 300 µL of hexane were added. The solution was moved to the prerinsed silica gel column. Then, the sample underwent elution with 6 mL of carbon tetrachloride, after which the eluate was dried using a rotary evaporator (Laborota 4000-efficient, Heidolph Instruments, Schwabach, Germany) and subsequently redissolved in 100 μL of hexane. Finally, 1 μL of the solution was injected into a gas chromatograph (Agilent Technologies 6890N). The operating conditions were as follows: the oven temperature program was set at 270 °C for 2 min, raised to 360 °C by 5 °C/min and maintained at 360 °C for 7 min; the PTV injector temperature program was 10 s at 60 °C, raised at 200 °C /min to 370 °C, and 3 min at 370 °C; the split ratio was 1:80; the FID temperature was 370 °C; the capillary column RTX-65 TG (Restek Corp., Bellefonte, PA, USA) has a 30 m × 0.25 mm i.d.; and a film thickness of 0.10 μm. The helium was used as a gas carrier at a flow of 2 mL/min.

#### 2.2.8. Sterol Determination

The sterol determination was carried out following the method reported by [55], with appropriate modifications. A 10% solution of fat in chloroform was prepared by carefully weighing 100 mg of fat and adding 1 mL of chloroform. The solution was vortexed for about 1 min. A total of 400 µL of potassium hydroxide (2 N) in methanol were added to the solution, everything was vortexed again for 1 min, and 800 µL of 1 N hydrochloric acid were then added. The solution was vortexed for 1 min and centrifuged at 4000 rpm for 5 min. The supernatant (1 µL) was collected and injected into an Agilent Technologies 6890N gas chromatograph equipped with a programmed temperature vaporizer (PTV), flame ionization detector (FID), and a capillary column (30 m × 0.25 mm i.d.; film thickness 0.10 μm) RTX-65 TG (Restek Corp., Bellefonte, PA, USA). The operating conditions were as follows: the PTV conditions were 80 °C for 0.1 min, increased by 500 °C/min up to 380 °C for 7 min; the oven temperature was set at 80 °C for 1 min, with an increase of 50 °C/min up to 280 °C for 15 min. The gas carrier was helium with a rated flow of 2 mL/min. The split ratio was 1:5. For the identification of the peaks, the retention times of the sample were compared with those of the external standard of sterols.

### 2.3. Determination of Elements and Contaminants

The detailed methodologies for analyzing essential, toxic, and rare earth elements, as well as polycyclic aromatic hydrocarbons (PAHs), can be found in the research conducted by [14]. Below is a brief overview of the reported procedures.

#### 2.3.1. Essential, Toxic, and Rare Earth Elements

A specific amount of 250 ± 1 mg for each PNS sample was digested in PP test tubes with ultrapure nitric acid and hydrofluoric acid. The digestion process was conducted in a preheated water bath, followed by the addition of boric acid and further digestion. The samples were then brought to a final volume using a 2% HNO_3_ solution for subsequent elemental analysis. An Aurora M90 ICP-MS instrument was used to analyze 25 elements (Ca, Mg, Na, K, Se, Zn, Fe, Cu, Mo, Al, As, Sb, Ba, Be, B, Cd, Co, Cr, Mn, Hg, Ni, Pb, Sn, Tl, and V), while sodium, potassium, magnesium, and calcium were analyzed separately using a 4210 MP-AES instrument. For each element, calibration curves were produced using certified standard solutions. To monitor contamination, reagent and digestion method blanks were utilized, and the calibration’s accuracy was confirmed through control standards. The limit of quantification (LOQ) was determined for each element, with varying ranges depending on the analysis method.

#### 2.3.2. Polycyclic Aromatic Hydrocarbons (PAHs)

For each sample, an aliquot of 1000 g ± 1 mg was extracted using a mixture of acetone and n-hexane in a 1:1 *v*/*v* ratio. The extraction process was carried out in closed glass vials using an ultrasonic bath for three cycles of 30 min each. Following extraction, the substance was concentrated using the MultiVap 8 automatic concentrator until it reached a final volume of 1 mL. The concentrated extract was then introduced into a gas chromatograph coupled with a mass spectrometer to identify the presence of 15 polycyclic aromatic hydrocarbons (PAHs). Calibration curves were established in the concentration range of 1 to 100 g/L by calculating the area ratio of the target ion to the corresponding internal standard using five standard solutions prepared from certified standards. The limit of quantification (LOQ) was established through the analysis of the blanks’ variability and was determined to be 0.05 mg/kg in the final sample. Calibration curves, controls, and the LOQ determination were used to ensure the accuracy and reliability of the analysis.

#### 2.3.3. Pesticides Residues

The dispersive SPE-modular QuEChERS (Quick Easy Cheap Effective Rugged Safe) method was used for acetonitrile extraction and the purification of pesticide residues from PNS according to the European standard [56]. The specific compounds analyzed are listed in Supplementary Table S1. For pesticide residue analysis, a multimethod approach combining GC-MS/MS and LC-MS/MS was employed. The limit of quantification (LOQ) for the analysis was established at 0.01 mg/kg. The entire analytical process was carried out in duplicate to ensure the accuracy and reliability of the results.

### 2.4. Nutraceutical Aspects

To assess the nutraceutical aspects, the isolation of proteins from pine nut skin and their subsequent analysis using high-resolution liquid chromatography-tandem mass spectrometry (HR LC-MS/MS) were conducted, following the methodology described in the study by [13].

Briefly, the PNS powder (10 g) was mixed with deionized water (100 mL) at a pH of 10.0, adjusted with 0.1 N of NaOH. After 4 h of stirring at 35 °C, a centrifugation was carried out at room temperature (25 °C) to collect the supernatants. To induce protein precipitation, the supernatants were acidified to a pH of 4.5 using 0.1 N of HCl and refrigerated overnight. The resulting protein precipitates were washed with cold acetone, dried, and dissolved in a trifluoroacetic acid (TFA) solution. The dissolved proteins were further purified using C-18 Sep-Pak cartridges and concentrated before being subjected to HR LC-MS/MS analysis. A Q-Exactive Orbitrap mass spectrometer coupled with an Ultimate 3000 ultra-high-performance liquid chromatography system was used for the analysis. The eluents consisted of formic acid in water and acetonitrile. The separated peptides were analyzed using a data-dependent acquisition mode, with precursor and fragmentation spectra acquired at specific settings. The acquired spectra were processed using Xcalibur Software and analyzed using ProteinProspector software (v 6.2.2. https://prospector.ucsf.edu/prospector/mshome.htm, accessed on 15 March 2024). The mass spectra were matched against a reference proteome database, and the potential bioactivity of the identified peptides was evaluated using Biopep software (https://biochemia.uwm.edu.pl/about-biopep-uwm, accessed on 15 March 2024). Uncharacterized protein sequences were subjected to a BLAST search to identify homologies with known proteins, and bioactive sequences with significant occurrences were reported for angiotensin-converting-enzyme (ACE) and dipeptidyl peptidase IV (DPP IV) inhibitory activities. 

### 2.5. Morphological-Structural Characterization

The pine nut skin samples underwent a detailed analysis through a range of analytical methods. Techniques such as Fourier Transform Infrared (FTIR) spectroscopy, solid-state CP/MAS 13C NMR spectroscopy, thermogravimetric analysis (TGA), scanning electron microscopy (SEM), and nitrogen adsorption volumetric analysis were utilized to examine the characteristics of the samples. For a complete understanding of the analytical techniques employed, readers are encouraged to refer to the study conducted by [13]. The original research provides detailed information on the specific procedures and equipment utilized for these analyses, offering a more in-depth exploration of the methods employed in the study.

### 2.6. Statistical Analysis

The LOQ was determined via a signal-to-noise ratio (S/N) of 10:1. The analysis of data and processing of graphs were conducted utilizing R Software version 3.6.0, along with the ggplot2 package [57]. Subsequently, the data underwent tests for variance homogeneity (Bartlett’s test) and normality (Shapiro–Wilk’s test). Finally, a one-way analysis of variance followed by a post hoc Tukey’s test was employed to determine any statistically significant differences.

## 3. Results

### 3.1. Nutritional Aspect

The pine nut skin (PNS), which is a by-product of the final stage in the pine nut production chain, has received limited attention. Therefore, a comprehensive analysis was conducted to understand its chemical composition and nutritional profile. The nutritional composition of PNS is primarily composed of carbohydrates (56.2%) and a significant amount of dietary fiber (27.5%). Total fat content accounted for 9.8%, and protein content was found to be 6.1% of the PNS.

Nuts are renowned for their high lipid content, which encompasses various bioactive compounds that contribute to health benefits [58]. Therefore, it is of interest to assess the lipid profile of their by-products. The results detailing the lipid composition and fatty acid profile of the PNS are presented in Table 1. Among the fatty acids identified, the PNS exhibited approximately 45% saturated fatty acids (SFAs) and 55% unsaturated fatty acids, comprising 35% monounsaturated fatty acids (MUFAs) and 20% polyunsaturated fatty acids (PUFAs). The most abundant fatty acids identified were palmitic (20%), oleic (35%), and linoleic acid (18%). Notably, oleic acid is recognized as a beneficial molecule in the prevention of various diseases, including autoimmune, metabolic, and cardiovascular diseases, as well as cancer, obesity, diabetes (type 2), and hypertension [59,60]. It is worth mentioning that the concentration of oleic acid in the PNS was similar to that found in pine nut oil [61]. The omega-6 fatty acid linoleic acid, which is crucial for physiological and metabolic functions and is linked to diseases like cardiovascular disease and cancer due to biologically active fatty acid mediators, was also detected at a concentration of 18.3% [62,63]. Another noteworthy fatty acid is eicosapentaenoic acid (EPA), which possesses health-promoting properties, although it was reported at a concentration of only 1.14% [64]. It is important to note that EPA is an essential omega-3 fatty acid that is rarely found in (non-transgenic) terrestrial plants [65].

However, if the omega-6/omega-3 ratio is high, this product should not be the sole fat source but rather included in a varied and balanced diet. Currently this matrix is a by-product, but future trends include its inclusion in food waste to functionalize it. Furthermore, the high concentration of unsaturated fatty acids (UFAs) confers a high fluidity and a low melting point to fat component [66], important factors to consider in food development. 

In conclusion, this waste, being rich in linoleic and oleic acids, which have been shown to counteract melanogenesis in cell culture and animal models [67], could be utilized in non-food applications.

This profile of fatty acids corresponded to a profile of triacylglycerols ranging from 48 to 54 carbon atoms. Triacylglycerols with 50 carbon atoms were dominant (10%), followed by C-52 triacylglycerols (9%). This composition was not similar to other seed oils, in which the main triacylglycerols were C-54 triacylglycerols [68]. The diglycerides, which varied from 38 to 46 carbon atoms were also abundant, representing 72% of the glyceride fraction. The wax content from pine nut skin was approximately 5%. This could find a correlation since waxes are the major component of the cuticle, which is a strong structural barrier that protects plant parts from the environment [69].

Finally, regarding sterol determination, the major one is beta-sitosterol (25.27 mg/g), followed by campesterol (2.33 mg/g), which is also present in the nut’s oils [70].

Phytosterols are plant-bioactive compounds with anticancer, antioxidant, anti-inflammatory, immunomodulatory, hepatoprotective, antimicrobial, anti-diabetic, sedative, analgesic, and lipid-lowering activity [71]. Notably, the consumption of 1.5–2.4 g/day of phytosterols is suggested to reduce serum cholesterol levels [72]. 

In a comparative analysis with a previous study [73], our examination of pine nut skin (PNS) revealed intriguing findings regarding its fatty acid composition. PNS exhibited a higher content of palmitic acid (C16:0) when compared to almond skin (8.36%), hazelnut skin (6.02%), and pistachio skin (8.94%). Similarly, PNS showed a higher content of stearic acid compared to these other nut skins. However, in terms of oleic acid (C18:1n9c), PNS displayed a lower content (35.33%) than hazelnut skin (76.53%), pistachio skin (44.42%), and almond skin (43.08%). Interestingly, the linoleic acid (C18:2n6c) content of PNS (18.34%) was moderate compared to almond skin (36.98%), pistachio skin (34.04%), and hazelnut skin (14.89%). These variations in fatty acid composition shed light on the unique nutritional profile of PNS, suggesting potential applications in various industries and nutraceutical formulations.

### 3.2. Occurrence of Essential Elements and Contaminants 

For a comprehensive characterization, the assessment of the principal essential elements in PNS was conducted. The most abundant element detected was K (4306 ± 294.16 mg/kg), followed by Na (1556 ± 181.02 mg/kg) and Mg (1485 ± 102.14 mg/kg) (Table 2). Other essential elements such as Ca (301 ± 8.42 mg/kg), Fe (244.73 ± 90.50 mg/kg), Zn (28.36 ± 17.55 mg/kg), Cu (11.10 ± 8.42 mg/kg), Mo (0.86 ± 0.20 mg/kg), and Co (0.12 ± 0.08 mg/kg) were also detected. Among the non-essential heavy metals, Al (55.6 ± 25.32 mg/kg) was reported with the highest concentration, followed by B (38.9 ± 7.02 mg/kg), Mn (25.06 ± 17.31 mg/kg), Ba (9.04 ± 2.09 mg/kg), Ni (1.36 ± 1.41 mg/kg), Hg (0.30 ± 0.11 mg/kg), As (0.14 ± 0.20 mg/kg), Cr (0.16 ± 0.15 mg/kg), and Cd (0.12 ± 0.18 mg/kg). However, Sb, Pb, Tl, Be, Sn, and V were reported in values below the limit of quantification (LOQ). 

Similarly, pesticide residues (Supplementary Table S1), and almost all rare earth elements (REE) (Table 2) and polycyclic aromatic hydrocarbons (PAHs) (Table 3) reported values < LOQ. Fluorene (0.12 ± 0.06 mg/kg) among the PAHs and La (0.06 ± 0.03 mg/kg), and Ce (0.08 ± 0.04 mg/kg) among REE were detected. Overall, most environmental contaminants were not found, but the occurrence of others could be attributed to the processing of PNS. Simple preliminary processing, such as washing, could reduce contaminants by removing particulate matter. However, attention should be paid to meeting hygienic standards for the reuse of PNS in the agri-food chain. Critical point control could be implemented if necessary to improve the safety profile of PNS.

### 3.3. Nutraceutical Aspect

The MS analysis revealed 19 peptides, primarily associated with proteins mainly involved in energy metabolism and defense mechanisms, including the legumin-like protein, class VII chitinase, and the heat shock 70 kDa protein (Hsp70) (Table 4 and Table 5). Among the uncharacterized proteins, A0A0L9TDU8 showed 90.3% homology with heat shock cognate 70 kDa protein-like (A0A1S3TT36). As an integral part of the metabolism of cells, Hsp70 folds and transports newly synthesized proteins across membranes into organelles, prevents protein aggregation, reassembles damaged proteins, and controls regulatory proteins [74]. It is likely that the presence of seed storage proteins, such as V9VGU0, Q41017, and Q40933, is due to the contamination of the integument with pine seed residuals.

Following the Biopep analysis of the peptides extracted from pine nut skin, a variety of bioactivities have been identified, as presented in Supplementary Table S2 and Supplementary Figure S1. The Biopep analysis highlighted the presence of precursor peptides that contain in their sequence fragments that show Angiotensin Converting Enzyme (ACE) inhibitory activities (i.e., SG, GD, GG, VR, HL, DA, GT, GE, LG, DF, and ST) and dipeptidyl peptidase IV (DPP IV) inhibitory activities (i.e., DN, TH, VR, AD, ES, GG, EP, NP, HL, GE, MN, NM, PS, and TS).

While present in a relatively small proportion, the peptides identified in the extracts also exhibit potentials as precursors for antioxidant sequences like HL and GGE, as well as alpha-glucosidase inhibitor peptides such as PE and AD. These bioactive sequences may be subject to release by hydrolytic enzymes during digestion in the gastrointestinal tract.

### 3.4. Morphological-Structural Characterization

The characterization of the chemical-physical and thermal features, as well as of the morphological structure of the PNS, opens the possibility of its reuse in the industrial sector. Over the last decade, this sector has been shifting towards a new sustainable model with the introduction of bio-based materials and waste recycling. The techniques utilized for the morphological-structural characterization of the PNS samples include Fourier Transform Infrared (FT-IR) spectroscopy, solid-state CP/MAS 13C NMR spectroscopy, thermogravimetric analysis (TGA), scanning electron microscopy (SEM), and the volumetric analysis of nitrogen adsorption. By employing these techniques, a comprehensive morphological-structural characterization of the sample can be achieved, aiding in the understanding of its properties and potential applications [75].

**Figure 1:**
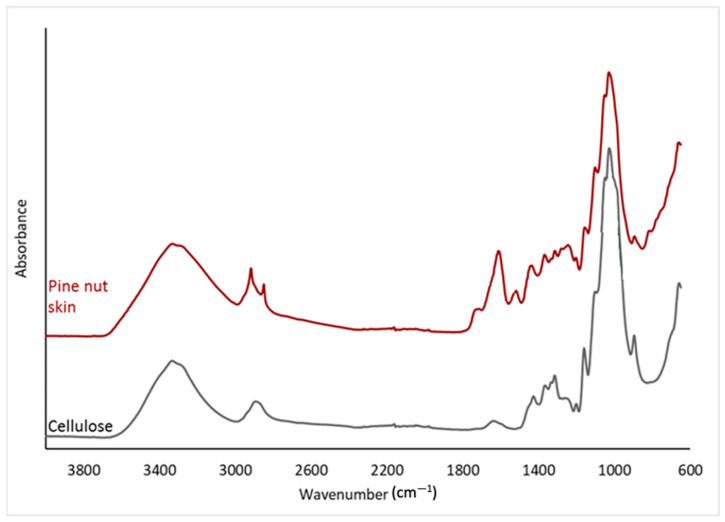
Fourier Transform Infrared (FT-IR) spectroscopy analysis was performed on two samples: pine nut skin (red) and a pure cellulose reference (black).

The FT-IR spectra of PNS (Figure 1) showed main complex absorption peaks centered at about 1000 cm^−1^, 1600 cm^−1^, and at 3350 cm^−1^. Moreover, overlapping peaks in the 1200–1450 cm^−1^ region, as well as a small shoulder at around 1700 cm^−1^ and a band at 2900 cm^−1^, are observed. The main features of the spectrum are strongly related to typical absorption peaks of the cellulose/hemicellulose, which are identified as the main PNS components. The obtained spectrum also displays additional bands in the aromatic ring and carbonyl vibration regions (1500–1750 cm^−1^), which are characteristics of the lignin fraction. Notably, the prominent absorption peak at approximately 1600 cm^−1^ is indicative of amide groups. Moreover, the presence of distinct and focused signals within the 2800–2900 cm^−1^ range (the C-H stretching region) is commonly observed in the spectra of lignin and hemicellulose. These results are consistent with earlier investigations [76]. Additionally, the intricate form of the carbonyl absorption band implies the existence of diverse carbonyl species. 

**Figure 2:**
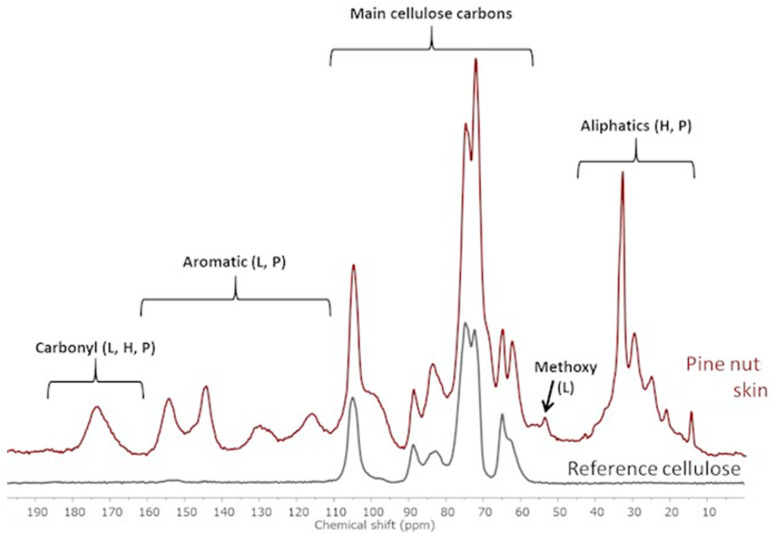
The Cross-Polarization/Magic Angle Spinning 13C Nuclear Magnetic Resonance spectra of pine nut skin (red) and a pure cellulose sample (black) are presented for comparison. A qualitative analysis has been performed to assign the main signals observed, indicating the presence of cellulose, lignin (L), hemicellulose (H), and proteins (Ps).

The PNS spectrum recorded in CP/ MAS 13C NMR mode (Figure 2) confirmed the identification of the main components of this integument as cellulose, hemicellulose, and lignin. The main carbon signals of polysaccharide structures (cellulose, hemicellulose) are observed in the range of 60–110 ppm. Additionally, the strong signals in the aromatic carbon region, ranging from 110 to 160 ppm, are primarily attributed to lignin. In the aliphatic region (10–40 ppm), a complex overlapping of signals can be observed: here, the occurrence of very narrow peaks is an indication of the presence of high mobility, low molecular weight species (e.g., waxes, oils), together with aliphatic chains that can be attributed to a peptide/protein fraction. Finally, the broad carbonyl signal, ranging from 165 to 180 ppm, can be attributed to both the amide carbonyls of proteins and the oxidized groups in lignin and hemicellulose.

A higher fiber content is common in protective seed covers, referring to the shells and the integument (mentioned as “skin”). The high fiber content of the skins has the advantage of stimulating food oxidation processes if it is used as a food ingredient [77], but in general, shells and skins can be classified as a natural lignocellulosic filler (NLF), for use in polymer matrix biocomposites [78,79].

**Figure 3:**
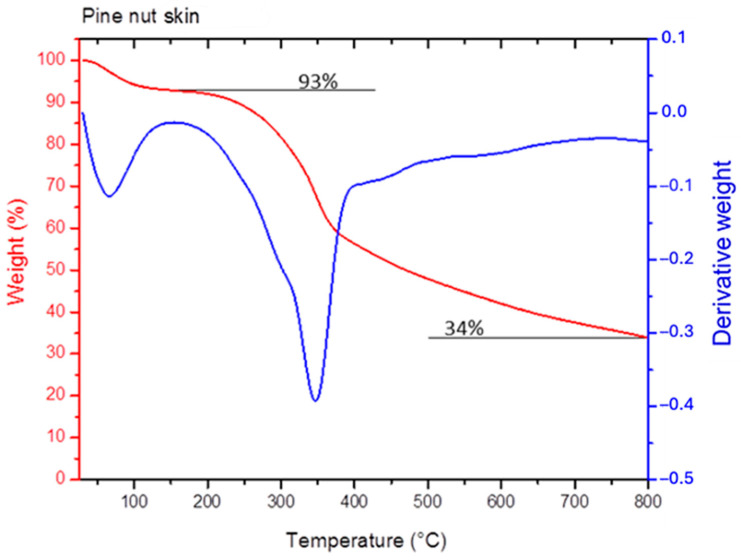
Thermogravimetric analysis and derivative weight loss curves of pine nut skin (PNS).

To investigate the degradation characteristics of PNS, a thermogravimetry analysis was conducted (Figure 3). The TGA curve exhibited three distinct mass loss stages. The initial stage, starting at 70 °C, involved the evaporation of surface-absorbed water, resulting in an approximately 7% weight loss. The second degradation stage, occurring between 190 and 400 °C, potentially encompassed various degradation processes (such as dehydration, decarboxylation, depolymerization, and decomposition) associated with the cellulose, hemicellulose, and lignin fractions. The maximum degradation rate was observed at 350 °C. The third and final degradation step was a gradual transition starting at around 380 °C with a lower degradation rate, resulting in a final char content of 34 wt%. This suggests that PNS has a high lignin/hemicellulose content, indicated by the relatively high char content and smooth appearance of the final degradation step.

**Figure 4:**
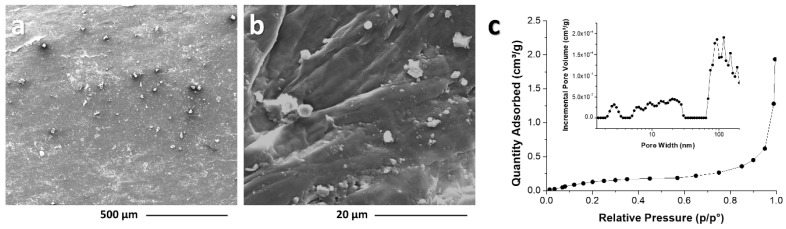
Scanning electron microscopy (SEM) images (**a**,**b**) and N_2_ adsorption isotherm and pore size distribution (**c**) of pine nut skin (PNS).

The scanning electron microscopy (SEM) analysis (Figure 4) provides visual insights into the structure of pine nut skin (PNS). Figure 4a reveals the surface of PNS, which appears as a compact and rugose surface, suggesting that the fibrous cellulose structure is embedded within a matrix composed of less structured hemicellulose/lignin components. In Figure 4b, the magnification allows for the observation of specific features, providing a closer look at the surface morphology of PNS. This analysis allows for the clear observation of cellulose fibers, measuring approximately 2.1–2.4 µm in thickness. Additionally, quasi-spherical particles ranging from 0.5–5 μm in size are observed on the surface of the PNS sheets, potentially originating from the lignin and/or protein fraction.

Subsequently, the N_2_ adsorption isotherm and pore size distribution analysis were both assessed to provide information about the porosity of the PNS. The PNS sample exhibits a type III isotherm, indicating low-pressure adsorption and predominantly macroporous characteristics. The isotherm curve illustrates the adsorption and desorption of nitrogen gas at different pressures, offering insights into the pore characteristics of the material. The corresponding pore size distribution chart provides additional details about the distribution of pore sizes within the PNS (Figure 4c). The pine nut skin demonstrates limited porosity, with a total pore volume of approximately 0.002 cm^3^/g and a BET specific surface area of about 0.71 ± 0.04 m^2^/g. These findings are consistent with the natural state of this type of natural resource.

Similar results were obtained from morphological-structural analyses on Coffee Silverskin [13], the integument recovered after the roasting phase and usually disposed of as waste. The literature reports that silverskin, as a lignocellulosic material, holds potential for reuse in the industrial sector [80,81]. Consequently, given the similarities between the structures and compositions of these integuments, PNS also has good prospects for industrial reuse.

## 4. Conclusions

In conclusion, this study sheds light on the potential of utilizing “skin” waste, exemplified by pine nut skin (PNS), within the framework of the circular economy (CE). Through a comprehensive analysis, including an examination of its chemical composition, nutritional profile, lipid composition, fatty acid profile, the occurrence of essential elements and contaminants, its nutraceutical aspect, and morphological-structural characteristics, valuable insights have been uncovered.

The nutritional analysis revealed that PNS is abundant in carbohydrates and dietary fiber, with moderate levels of protein and fat. Moreover, the lipid composition showcased a predominance of beneficial unsaturated fatty acids, notably oleic acid, while the presence of essential omega-3 fatty acids like eicosapentaenoic acid (EPA) hints at potential health benefits. Additionally, the identification of bioactive compounds such as phytosterols and peptides associated with various metabolic pathways further enhances the nutraceutical value of PNS. Furthermore, its morphological structural characterization unveiled a complex composition, featuring cellulose fibers embedded within a matrix of hemicellulose and lignin components. The nitrogen adsorption isotherm analysis emphasized the predominantly macroporous nature of PNS.

Overall, the comprehensive characterization of PNS underscores its potential for industrial reuse in sustainable applications across diverse sectors. However, further studies are warranted to explore avenues for enhancing its fatty acid content, fiber, and other beneficial compounds, as well as exploring synergies with different types of skins. This study on pine nut skin (PNS) acknowledges limitations such as the limited prior research, potential sample variability, and uncertainties in environmental contamination. While comprehensive analyses reveal its nutritional composition, lipid profile, and bioactivity, variations in results and comparisons with other materials highlight the need for further validation studies to enhance reliability and the understanding of PNS characteristics for potential industrial reuse. Moreover, parallel advancements in sustainable processing technologies, along with appropriate storage and packaging solutions, are imperative to ensure the efficient management and utilization of these “new resources” while mitigating potential contamination risks during accumulation phases.

## Figures and Tables

**Table 1:** Lipid composition and fatty acid profile (%) of pine nut skin samples.

Fatty Acid	Values (%)
Palmitic acid (C16:0)	19.98 ± 0.22
Stearic acid (C18:0)	6.06 ± 0.28
Oleic acid (C18:1n9c)	35.33 ± 0.28
Linoleic acid (C18:2n6c)	18.34 ± 0.82
Arachidic acid (AA) (C20:0)	10.77 ± 0.60
Tricosanoic acid (C23:0)	8.38 ± 0.50
Eicosapentaenoic acid (EPA) (C20:5n3)	1.14 ± 0.04
∑SFA	45.19
∑MUFA	35.33
∑PUFA	19.48
Omega 6/Omega 3	16.11

**Table 2:** Essential, toxic elements, and rare earth elements (REE) in pine nut skin samples. The values are presented as mean (*n* = 3) ± standard deviation. Values below the limit of quantification are indicated as <LOQ for each category.

Essential, Toxic, and Rare Earth Elements	Mean ± SD Concentration (mg/kg)
Calcium, Ca	301 ± 8.42
Magnesium, Mg	1485 ± 102.14
Sodium, Na	1556 ± 181.02
Potassium, K	4306 ± 294.16
Selenium, Se	<LOQ
Cobalt, Co	0.12 ± 0.08
Iron, Fe	244.73 ± 90.50
Zinc, Zn	28.36 ± 17.55
Copper, Cu	11.10 ± 8.42
Chromium, Cr	0.16 ± 0.15
Cadmium, Cd	0.12 ± 0.18
Molybdenum, Mo	0.86 ± 0.20
Antimony, Sb	<LOQ
Boron, B	38.9 ± 7.02
Arsenic, As	0.14 ± 0.20
Manganese, Mn	25.06 ± 17.31
Mercury, Hg	0.30 ± 0.11
Nickel, Ni	1.36 ± 1.41
Lead, Pb	<LOQ
Beryllium, Be	<LOQ
Aluminum, Al	55.6 ± 25.32
Tin, Sn	<LOQ
Thallium, Tl	<LOQ
Vanadium, V	<LOQ
Barium, Ba	9.04 ± 2.09
Yttrium, Y	<LOQ
Lanthanum, La	0.06 ± 0.03
Cerium, Ce	0.08 ± 0.04
Praseodymium, Pr	<LOQ
Neodymium, Nd	<LOQ
Samarium, Sm	<LOQ
Europium, Eu	<LOQ
Gadolinium, Gd	<LOQ
Terbium, Tb	<LOQ
Dysprosium, Dy	<LOQ
Holmium, Ho	<LOQ
Erbium, Er	<LOQ
Thulium, Tm	<LOQ
Ytterbium, Yb	<LOQ
Lutetium, Lu	<LOQ

**Table 3:** Polycyclic aromatic hydrocarbons (PAHs) in pine nut skin samples. The values are presented as mean (n = 3) ± standard deviation. Values below the limit of quantification are indicated as <LOQ for each category.

Polycyclic Aromatic Hydrocarbons	Mean ± SD Concentration (mg/kg)
Naphthalene	<LOQ
Acenaphtilene	<LOQ
Acenaphthene	<LOQ
Fluorene	0.12 ± 0.06
Anthracene	<LOQ
Phenanthrene	<LOQ
Fluoranthene	<LOQ
Pyrene	<LOQ
Chrysene	<LOQ
Benzo(b)fluoranthene	<LOQ
Benzo(k)fluoranthene	<LOQ
Benzo(a)pyrene	<LOQ
Indeno[1,2,3-cd]pyrene	<LOQ
Dibenz[a,h]anthracene	<LOQ
Benzo[ghi]perylene	<LOQ

**Table 4:** Proteins and peptides identified in pine nut skin (PNS). Their corresponding Uniprot IDs were obtained from the UniprotKB protein database (www.uniprot.org (accessed on 15 March 2024)). The expectation value (E-value) associated with each identification serves as a score reflecting the confidence level of the peptide/protein identification.

Uniprot ID	E-Value	Peptides	Species	Protein Name
V9VGU0	6.70 × 10^−9^	4	*Pinus koraiensis*	Vicilin Pin k 2.0101
Q41017	7.10 × 10^−11^	3	*Pinus strobus*	Pine globulin 1
A0A103XGC0	3.70 × 10^−8^	1	*Cynara scolymus*	Aspartate aminotransferase
A0A5C0ZT38	3.30 × 10^−8^	1	*Pinus banksiana*	Class VII chitinase
O65719	3.60 × 10^−10^	1	*Arabidopsis thaliana*	Heat shock 70 kDa protein 3
P50218	1.30 × 10^−8^	1	*Nicotiana tabacum*	Isocitrate dehydrogenase [NADP]
Q40933	5.80 × 10^−9^	1	*Pseudotsuga menziesii*	Legumin-like storage protein
A0A067KQG0	1.90 × 10^−10^	1	*Jatropha curcas*	LNS2 domain-containing protein
Q40924	4.10 × 10^−9^	1	*Pseudotsuga menziesii*	Luminal binding protein
Q6S3D6	1.20 × 10^−9^	1	*Populus tomentosa*	Phosphoglucomutase
O04386	8.30 × 10^−8^	1	*Chlamydomonas incerta*	Tubulin beta chain
A0A0L9TDU8	4.40 × 10^−10^	1	*Phaseolus angularis*	Uncharacterized protein
A0A2G2XQ03	1.90 × 10^−10^	1	*Capsicum baccatum*	Uncharacterized protein
A0A6A6K508	1.90 × 10^−10^	1	*Hevea brasiliensis*	Uncharacterized protein

**Table 5:** Peptides found in the aqueous extract of pine nut skin protein isolate. The expectation value (E-value) associated with each identification serves as a score reflecting the confidence level of the peptide/protein identification.

Uniprot ID	Peptide	Start	End	E-Value	Species	Protein Name
A0A067KQG0	IPDEMNM	813	819	1.90 × 10^−10^	JATCU	LNS2 domain-containing protein
A0A0L9TDU8	ATAGDTHLGGEDIDNR	165	180	4.40 × 10^−10^	PHAAN	Uncharacterized protein
A0A103XGC0	ASGSLDQDASSVR	234	246	3.70 × 10^−8^	CYNCS	Aspartate aminotransferase
A0A2G2XQ03	IPEDMNM	93	99	1.90 × 10^−10^	CAPBA	Uncharacterized protein
A0A5C0ZT38	SGFGTTGTSDDNKRELA	66	82	3.30 × 10^−8^	PINBN	Class VII chitinase
A0A6A6K508	LPDEMMN	643	649	1.90 × 10^−10^	HEVBR	Uncharacterized protein
O04386	MDLEPGTMDSVR	66	77	8.30 × 10^−8^	CHLIN	Tubulin beta chain
O65719	ATAGDTHLGGEDFDNR	227	242	3.60 × 10^−10^	ARATH	Heat shock 70 kDa protein 3
P50218	IKVENPIVEMDGDEMTR	6	22	1.30 × 10^−8^	TOBAC	Isocitrate dehydrogenase [NADP]
Q40924	STSGDTHLGGEDFDQR	263	278	4.10 × 10^−9^	PSEMZ	Luminal binding protein
Q40933	HNADNPEDADIYVR	328	341	5.80 × 10^−9^	PSEMZ	Legumin-like storage protein
Q41017	LSTHEPSESESIR	26	38	3.00 × 10^−8^	PINST	Pine globulin 1
Q41017	HNADNPEDADVYVR	300	313	4.00 × 10^−8^	PINST	Pine globulin 1
Q41017	LSTHEPSESESIRSE	26	40	7.10 × 10^−11^	PINST	Pine globulin 1

## Data Availability

The original contributions presented in the study are included in the supplementary material, further inquiries can be directed to the corresponding author.

## Supplementary Materials

The following supporting information can be downloaded at: https://www.mdpi.com/article/10.3390/foods13071044/s1, Table S1: Multiresidual list of pesticides assessed in pine nut skin (PNS) by GC-MS/MS (list 1) and LC-MS/MS (list 2); Table S2: Bioactivity mapping; Figure S1: Graphical representation of the main bioactive sequences found in SPI peptides. The bioactive sequences were grouped per bioactivity. The graph was made using ggplot2 package in R (https://ggplot2.tidyverse.org.ggplot2 (accessed on 4 July 2023)).
