# Supplementary material for: Exploring the Untapped Potential of Pine Nut Skin By-Products: A Holistic Characterization and Recycling Approach
Source: Foods. 2024 Mar 28;13(7):1044. doi: 10.3390/foods13071044 (PMC11011278; doi:10.3390/foods13071044)
Supplement: Supplementary file 1 [file foods-13-01044-s001.zip › Table S1.pdf]

Table S1. Multiresidual list of pesticides assessed in pine nut skin (PNS) by GC-MS/MS (list 1) and LC-MS/MS (list 2)

LIST 1

Acephate	Aclonifen	Acrinathrin	Alachlor
Ametryn	Amitraz	Atrazine	Azinphos ethyl
Azinphos methyl	Azoxystrobin	Benalaxyl	Benfluralin
Benfuracarb	Bifenthrin	Bitertanol	Boscalid
Bromopropylate	Bromuconazole (sum of	Bupirimate	Buprofezin
Cadusafos	diastereoisomer)	Captan	Carbaryl
Carbofuran	Captafol	Carbosulfan	Chiorfenapyr
Chiorfevinphos	Carbophenothion	Chlorothalonil	Chlorpropham
Chiorpyriphos ethyl	Chlormefos	Chlorthal dimetil	Chlozolinate
Chlordane	Chilorpyriphos methyl	Cyproconazole	Cyprodinil
Cyflutrin (sum of isomers)	Cypermethrin (sum of isomers)	Diazinon	Dichlobenil
DDT (sum of p,p' DDT, o,p'	Deltamethrin	Dichlorvos	Diclobutrazol
DDT, p,p' DDE, and p,p' TDE	Dichloran.	Difenoconazole	Dimethoate (sum of Dimethoate
(DDD) expressed as DDT)	Dieldrin (sum of Aldrin	Endosulfan-sulfate (sum of	and Omethoate expressed
Dichiofluania	and Dieldrin expressed	isomer a,b, e sulfate	as Dimethoate)
Dicofol	as Delrin)	expressed as Endosulfan)	Diquat
Diphenylamine	Disulfoton	Ethion	Endrin
Esfenvalerate	Ethiofencarb	Exithiazox	Ethoprophos
Ethoxyguine	Etofenprox	Fenarimol	Famoxadone
Fenamidone	Fenamiphos	Fenhexamid	Fenazaquin
Fenbuconazole	Fenchlorphos	Fenpropidin	Fenitrothion
Fenoxycarb	Fenpropathrin	Fluazifop P.butile	Fenthion
Fenvalerate	Fipronil	Fluvalinate	Flucytrinate
Fludioxonl	Flusilazole	Heptachlor (sum of	Folpet
Furalaxit	Furathiocarb	Heptachlor and Heptachlor	Heptenophos
Hexachlorobenzene	HCH-a	epoxide expressed as Heptachlor)	Gamma - hexachorohexane (lindane)
Hexaconazole	haloxyfop methyl	HCH	Iprodione
Iprovalicarb	Imazall	Indoxacarb (sum of Indoxacarb	Lambda-Cyhalothrin
Lenacil	Isophenphos	and its enantiomer R)	Mepanipyrim
Metalaxyl (sum of isomers	Linuron	Kresoxim-methyl	Methiocarb
including Metalaxyl-m)	Methamidophos	Malathion	Metribuzin
Metholachlor	Methomyl	Methidathion	Oxamyl
Myclobutanil	Nuarimol	Methoxiclor	Parathion methyl
Oxyfluorfen	Paclobutrazolo	Oxadixyl	Pertane
Penconazole	Pendimetalin	Parathion ethyl	Phosphamidone
Phorate	Phosalone	Permethrin (sum of isomers)	Profenophos
Pirimicarb	Prochloraz	Phosmet	Propham
Propachior	Propamocarb	Procymidone	Pyraciostrobin
Propiconazole	Propoxur	Propargite	Pyridaphenthion
Pyrazofos	Pyrethrins (technical mixture)	Propyzamide	Quinalfos
Pyrifenox	Pyrimethanil	Pyridaben	Simazine
Quinoxifen	Quintozene	Pyriproxyfen	Tebuconazole
Symetryn	Spiromesifen	Quizalofop ethyl	Terbutryn
Tebutenpyrad	Tecnazen	Sulfotep	Tetramethrin
Tetrachlorvinphos	Tetraconazole	Tefluthrin	Triadimefon e Triadimenol (sum of
Thionazin	Tolclofos methyl	Tetradifon	Triadimefon and Triadimenol)
Triazophos	Trichlorfon	Tolylfluanid	Trifluralin
Vinclozolin	Zoxamide	Trifioxystrobin	

LIST 2

2,4-D (sum of 2,4-D, its	Abamectin	Acephate	Acetamiprid
salts, esters, and conjugates	Ametoctradin	Atrazine	Azadirachtin
expressed as 2,4-D)	Benthiocarb	Bifenazato (sum of	Boscalid
Aldicarb (sum of Aldicarb,	Bromadiolone	bifenazato and	Butoxycarboxim
Aldicarb- sulfone, and	Carbaryl	bifenazato- diazene	Carbofuran (sum of
Aldicarb- sulfoxide	Chlorbromuron	expressed as bifenazato)	Carbofuran and 3-
expressed as Aldicarb)	Clethodim	Bromuconazole (sum of	Hydroxycarbofuran
Azoxystrobin	Cyazofamid	Diastereoisomeri)	expressed as Carbofuran)
Brodifacoum	Cyromazine	Carbendazim (sum of	Chlorsulfuron
Buturon	Difenoxuron	Benomil and Carbendazim	Cyanophenfos
Chlorantraniliprole	Diniconazole	expressed as Carbendazim)	Cyflufenamid
Chlothianidin	Dodine	Chlorotoluron	Dichlofluanid
Cyantraniliprole	Etiofencarb	Clofentezine	Diflufenican
Cymoxanil	Fenazaquin	Cycloxydim	Ditalimfos
Diethofencarb	Fenoxycarb	Demeton S-methyl sulphone	Emamectina benzoato
Dimethomorph	Fenuron	Diflubenzuron	(Emamectina B1A expressed
Diuron	Fluometuron	Dioxacarb	as Emamectina)
Epoxiconazole	Flutriafol	Ema B1B	Etoxazole
Famoxadone	Furathiocarb	Ethofenprox	Fenbutatin oxide
Fenhexamid	Imazamox	Fenbuconazole	Fenpyrazamine
Fenpyroximate	Isocarbophos	Fenpropimorph	Flonicamid (sum of
Flufenoxuron	Linuron	Fipronil (sum of Fipronil	Flonicamid, TFNA, and TFNG

Flupyradifurone
Fosthiazate
Imazalil
Iprovalicarb
Isopyrazam
Mepanipyrim
Metalaxyl-m Methiocarb (sum of Methiocarb, Methiocarb- sulfone, Methiocarb-sulfoxide expressed as Methiocarb)
Metobromuron
Milbemectina A4
Nitenpyran
Oxamyl-oxime
Pirimicarb
Prometrin
Prosulfocarb
Pyriproxyfen
Spinetoram
(Spirotetramat and its 4 metabolites expressed as Spirotetramat)
Tebufenpyrad
Thiametoxam
Tolclofos methyl
Zoxamide

Mepronil
Metamitron
Metholachlor, S-
Metoxuron
Monolinuron
Oxadiazon
Oxydemethon methyl
Pirimiphos-ethyl
Propamocarb (sum of Propamocarb e its salts expressed as Propamocarb)
Pymetrozine
Rotenone
Spinosad (sum of Spinosyn-a, Spinosyn-d expressed as Spinosad)
Spiroxamine
Teflubenzuron
Thiobencarb
Tridemorph
(Aquatecide) 2-4 Na diclorophenoxyacetate
Tiguron

and Sulfone metabolite expressed as Fipronil)
Fluopicolide
Fluxapyroxad
Hexaflumuron
Imidacloprid
lsofenphos-methyl
Lufenuron Metaflumizone
Metazachlor
Methomyl (sum of Methomyl and Thiodicarb expressed as Methomyl)
Metrafenone
Monuron
Oxadixyl
Penthiopyrad
Pirimiphos-methyl
Propargite
Pyraclostrobin
Simazine
Spirodiclofen
Sulfoxaflor (sum of isomers)
Thiabendazole
Thiocyclam
Triflumuron

exoressed as Flonicamid)
Fluopyram Formetanate
Hexythiazox
Indoxacarb (sum of indoxacarb and its enantiomer R)
lsoproturon
Mandipropamid
Metalaxyl (sum of isomers including Metalaxyl-m)
Methamidophos
Methoxyfenozide
Milbemectina A3
Neburon
Oxamyl
Phenmedipham
Promecarb
Propoxur
Pyraflufen-ethyl
Milbemectina (sum of Milbemectina A4 and Milbemectina A3 expressed as Milbemectina
Spiromesifen
Tebufenozide
Thiacloprid
Thiophanate methyl
Triforine
